# Quercetin and Related Analogs as Therapeutics to Promote Tissue Repair

**DOI:** 10.3390/bioengineering10101127

**Published:** 2023-09-25

**Authors:** Tina B. McKay, Kyle A. Emmitte, Carrie German, Dimitrios Karamichos

**Affiliations:** 1 Department of Anesthesia, Critical Care and Pain Medicine, Massachusetts General Hospital, Boston, MA 02114, USA; tmckay@mgh.harvard.edu; 2 Department of Pharmaceutical Sciences, UNT System College of Pharmacy, University of North Texas Health Science Center, Fort Worth, TX 76107, USA; kyle.emmitte@unthsc.edu; 3 CFD Research Corporation, Computational Biology Division, Huntsville, AL 35806, USA; carrie.german@cfdrc.com; 4 North Texas Eye Research Institute, University of North Texas Health Science Center, Fort Worth, TX 76107, USA; 5 Department of Pharmacology and Neuroscience, School of Biomedical Sciences, University of North Texas Health Science Center, Fort Worth, TX 76107, USA

**Keywords:** flavonoids, fibrosis, cornea, quercetin, wound healing, aging, neuroprotective, kaempferol, myricetin

## Abstract

Quercetin is a polyphenol of the flavonoid class of secondary metabolites that is widely distributed in the plant kingdom. Quercetin has been found to exhibit potent bioactivity in the areas of wound healing, neuroprotection, and anti-aging research. Naturally found in highly glycosylated forms, aglycone quercetin has low solubility in aqueous environments, which has heavily limited its clinical applications. To improve the stability and bioavailability of quercetin, efforts have been made to chemically modify quercetin and related flavonoids so as to improve aqueous solubility while retaining bioactivity. In this review, we provide an updated overview of the biological properties of quercetin and proposed mechanisms of actions in the context of wound healing and aging. We also provide a description of recent developments in synthetic approaches to improve the solubility and stability of quercetin and related analogs for therapeutic applications. Further research in these areas is expected to enable translational applications to improve ocular wound healing and tissue repair.

## 1. Introduction

The earliest evidence for the use of medicinal plants in archaic human history dates back to the Paleolithic period by the Neanderthals nearly 60,000 years ago [1,2,3]. In modern times, natural products and their modified semisynthetic forms remain a steady source of new therapeutics to treat disease, and about one-quarter of natural products approved by the Food and Drug Administration in the United States are plant-derived [4,5]. Quercetin (3,5,7,3′,4′-pentahydroxyflavone) is a polyphenol flavonoid derived from plants and legumes that exhibits a diverse range of bioactivity. Originally derived from the Latin word *quercetum*, meaning oak-wood [6], quercetin is a small molecule with a distinctive yellow color that possesses potent antioxidant properties as a reactive oxygen species (ROS) scavenger. Quercetin and other flavonoids are abundant in apples, grapes, plums, coriander, corn poppy, and fennel, and enriched in the leaves of onion, thale cress, olives, and sweet potatoes, among other fruits and vegetables [7,8,9]. Quercetin content in onion leaves is estimated at ~1500 mg/kg dry weight of plant material [8]. The biosynthetic pathways involved in flavonoid synthesis have been extensively reviewed in the literature [10,11,12]. Following exposure to ultraviolet light (UV)-B, quercetin levels significantly increase in the leaves of *C. sativum* (coriander) and other plants, and its production, in concert with various polyphenols, is thought to serve a functional role against ROS-induced damage promoted by UV activation [13,14]. This adaptive dose-dependent response, termed hormesis, has been proposed as a protective mechanism present in plants that involves increased release of flavonoids in response to exogenous stressors that may contribute to enhanced resilience to further high-dose cytotoxins [15]. The beneficial properties of quercetin and related flavonoids is largely conserved in the animal kingdom and thought to be a form of ‘xenohormesis,’ in which protective agents derived from one domain (plants) are beneficial to another organism (humans) via shared survival mechanisms but independent biological pathways [16]. 

Perhaps the most well-studied therapeutic property of quercetin is direct quenching of ROS to a less reactive chemical species, as well as chelation of exogenous reactive metals that may also promote the generation of excess ROS [17,18]. Recent work has highlighted other downstream pathways modulated by quercetin, including activation of the sirtuin family of proteins [19]. Sirtuins are deacetylases that regulate regenerative responses in the cell and are activated by exogenous stressors, such as extreme cold, hypoxia, and nutrition restriction [20,21,22]. Quercetin supplementation has been associated with activation of these pro-reparative pathways and favorable neuroprotective and anti-aging properties in animal models [23,24]. Quercetin is also a phytoestrogen, a known agonist of estrogen receptor α and β, by which it may also influence downstream hormonally-regulated pathways involved in cell proliferation [25].

Due to its potent biological properties, further developments to improve the bioavailability of quercetin and related analogs have gained substantial interest in recent years. In this review article, we provide an updated overview of the biological properties of quercetin in the context of wound healing with a focus on ocular applications; we also discuss current approaches to improve bioavailability via chemical modification. Further study of quercetin and related analogs is expected to accelerate therapeutic applications and enable broad utilization of these natural products to improve human health.

## 2. Bioactivity

Many of the health benefits associated with consuming green leafy plants have been at least partly attributed to the presence and bioactivity of quercetin and related flavonoids [26,27,28]. As anecdotal evidence, onion extract has long been proposed as a natural treatment for scarring and burns [29,30], and onions are known to be composed of moderate amounts of quercetin that can be absorbed into the bloodstream following oral consumption [31,32]. Oral bioavailability of quercetin via food derivatives occurs with a blood distribution of 0–0.44 μM/L in the plasma of 3′-methyl quercetin following a single intake of ~1 L of apple cider [33]. While high-dose intake of quercetin is thought to be generally safe, the therapeutic effectiveness of quercetin administered orally is largely limited by its low bioavailability [34]. Quercetin is highly glycosylated in its natural form with reduced absorption as an aglycone. Structural and functional analyses of quercetin have shown a relationship between hydroxylation of quercetin at C^3^, C^5^, C^7^, C^3′^, and C^4′^ and increased antioxidant capabilities [35]. Preclinical studies in animal models have suggested that quercetin has potent antifibrotic, antioxidative, and anti-inflammatory properties in the anterior segment of the eye that favors tissue repair and maintenance (Table 1). In this section, we review the biological effects of quercetin in the context of wound healing and aging with a focus on ocular implications.

### 2.1. Wound Healing 

Preclinical studies have revealed promising results suggesting that quercetin treatment may inhibit fibrosis in different tissues. We have found that topical application of quercetin to the corneal surface appears to lead to a moderate reduction in scarification in mice and rabbits following epithelial debridement and penetrating keratectomy, respectively [36]. Studies in the skin have shown similar findings with less scarring observed with quercetin application following burn exposure or wounding [41,42], which has also been proposed as a potential preventative treatment for the formation of keloids [43]. In diabetic animal models that are commonly associated with decreased wound healing capabilities, application of quercetin has shown improved skin closure following laceration and chronic wound management [44,45]. The antioxidative properties of quercetin also appear to extend to protection against diabetes-associated cataract formation in animal models of type I diabetes [37,38]. Moreover, topical ocular application of quercetin and resveratrol have been associated with decreased proinflammatory cytokine tear secretion and improved ocular manifestations in mouse models of dry eye disease [39,40], suggesting that quercetin may also influence inflammatory processes that affect the ocular surface. 

In terms of the mechanisms underlying the observed antifibrotic properties, quercetin has been found to be a potent metabolic regulator at both the individual cell level [46] and the tissue level in the liver [47], leading to systemic effects on glucose and lipid distributions [48]. Studies in the lung have identified increased lactate production, lower pH in the extracellular space, and myofibroblast differentiation in fibrotic tissue [49,50], and this process may be collectively inhibited by quercetin treatment via modulation of the redox component [51,52]. We have found that quercetin stimulation reduces lactate production and expression of α-smooth muscle actin (α-SMA) and collagen type III in human corneal fibroblasts cultured in vitro [53]. Altered metabolite levels and energy metabolism via the tricarboxylic acid cycle has been observed in corneal fibroblasts derived from patients with keratoconus [54], a corneal thinning disease associated with altered extracellular matrix deposition in the corneal stroma, and quercetin treatment has been found to influence this metabolic phenotype in concert with decreased expression of fibrotic markers [53,55]. The metabolic effects of quercetin extend to cancer cell types, with a similar reduction in lactate production observed with quercetin treatment [56], and is thought to inhibit the Warburg metabolic phenotype [57]. The Warburg effect is a metabolic change in which energy production via aerobic glycolysis is favored over the high-demand tricarboxylic acid cycle, which can be cumbersome in highly proliferative cell states, such as cancer [58].

The biological activity of quercetin has also been attributed to regulation of the acute immune response involving macrophages in the wounded area, whereby modulation of the proinflammatory response appears to ameliorate tissue damage that may be promoted by excessive ROS production by invading immune cells [44]. Quercetin has also been found to reduce expression of proinflammatory cytokines directly, including tumor necrosis factor-α (TNF-α), interleukin (IL)-1β, and IL-6 in concert with increased canonical Wnt-signaling in the skin of wounded mice treated with quercetin [59]. While the immune response is essential in preventing infection, targeted and controlled regulation of inflammation by small molecules, such as quercetin, may inhibit scar formation in a similar process to anti-inflammatory treatments [60]. Moreover, the antioxidative properties of quercetin extend to its upregulation of protective enzymes responsible for quenching ROS, including superoxide dismutase (SOD) 1 and 2 and catalase in an atopic dermatitis model [61]. Quercetin has also been reported as an inhibitor of angiogenesis via regulation of the vascular endothelial growth factor pathway in retinal models [62,63]. While these preclinical studies have highlighted the broad biological activity of quercetin, double-blinded placebo-controlled studies in human populations are still needed to establish the therapeutic benefits of quercetin application in ocular wound healing. 

### 2.2. Aging

**Figure 1:**
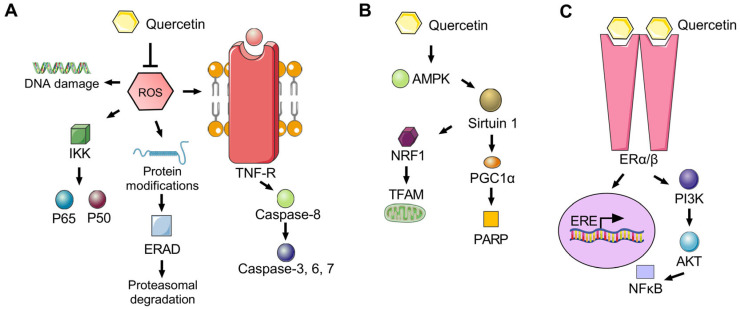
Selected downstream signaling pathways important in the bioactivity of quercetin. (**A**) Antioxidative properties of quercetin may influence downstream signaling pathways mediated by reactive oxygen species (ROS). (**B**) Quercetin may influence mitochondrial function via activation of Sirtuin 1 and mitochondrial-associated genes. (**C**) As a phytoestrogen, quercetin is able to bind to membrane-bound estrogen receptors or cytosolic estrogen receptor α or β and influence downstream signaling. (Abbreviations: protein kinase B (AKT); 5′-adenosine monophosphate–activated protein kinase (AMPK); estrogen receptor (ER)α/β; endoplasmic reticulum associated protein degradation (ERAD); estrogen response element (ERE); inhibitor of NF-κB kinase (IKK); nuclear respiratory factor 1 (NRF1); poly (ADP-ribose) polymerase (PARP); peroxisome proliferator–activated receptor-gamma coactivator (PGC1α); transcription factor A mitochondrial (TFAM); and tumor necrosis factor-receptor (TNF-R)). Based on references [71,72,73,74].

The biological markers of aging are highly conserved throughout the animal kingdom from yeast to humans. Changes associated with aging include deficiencies in genomic stability, telomere length, nutritional sensing, and mitochondrial function, among others [64] that collectively determine cell, tissue, and organismal survival. Downstream effectors influenced by quercetin treatment include its ROS-scavenging properties and mediators of autophagy, among other pathways (Figure 1). As a potent antioxidant, quercetin may inhibit ROS-driven processes that are associated with DNA damage, activation of the inhibitor of the NF-κB kinase (IKK) signaling pathway, induction of protein modifications and proteasomal degradation, and tumor necrosis factor-receptor (TNF-R)-induced apoptosis. The pleiotropic properties of quercetin include its potent antioxidative properties, as well as its functional effects on sirtuin signaling and mitochondrial function mediated via activation of 5′-adenosine monophosphate-activated protein kinase (AMPK). Moreover, many of the biological effects of quercetin and resveratrol in terms of aging and longevity have been at least partly attributed to their influence on sirtuin activation [24,65,66]. The sirtuin family of proteins are deacetylases important in DNA repair, nutritional responses, and cell metabolism. Sirtuin 1 is an isoform associated with histone deacetylation that is particularly important in mitochondrial biogenesis, and inhibition of Sirtuin 1 via chemical blocking leads to widespread increases in the acetylation patterns of proteins predominately found within the mitochondria, thus highlighting its important role in mitochondrial function [67]. Sirtuin 1 is an activator of peroxisome proliferator-activated receptor γ coactivator-1α (PGC-1α), which is also responsive to exercise and nutrition [68]. Quercetin has been found to promote a 5-fold increase in Sirtuin 1 activity in yeast [24], with up to a 60% increase in lifespan observed [69]. 

In terms of other pathways influenced by quercetin, activation of estrogen receptor β and the estrogen response element by quercetin stimulation appear to be comparable or even higher than estradiol in vitro, which may influence cell proliferation [25,70]. While the hormonal properties of quercetin as a phytoestrogen have focused heavily on cancer cell types, the wound healing properties of quercetin observed in the epidermis and corneal stroma may also be related to its activation of estrogen receptor–mediated signaling. Further mechanistic studies of quercetin and related analogs may aid in identifying the prime molecular targets involved in its antifibrotic properties and enable more targeted therapeutic development.

## 3. Therapeutic Development

**Figure 2:**
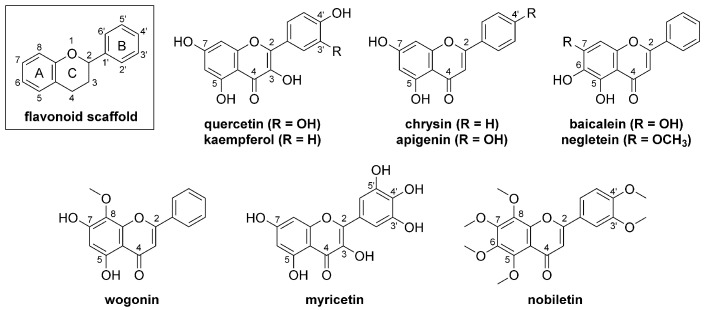
Quercetin and structurally related flavonoid natural products.

Flavonoids consist of a chromane ring (A- and C-rings) substituted by a phenyl ring (B-ring) at C^2^ (Figure 2). Quercetin and structurally similar natural products maintain an unsaturated bond at C^2^-C^3^ and a ketone at C^4^ of the C-ring and are a part of the flavone subclass of flavonoids. Variation between these compounds primarily relates to the number and position of the phenolic hydroxyl groups, which may be methylated in some instances [75]. Each of these compounds is highly aromatic and lacks ionizable functional groups at physiologically relevant pH values, which contributes to their aforementioned low aqueous solubility and bioavailability [34]. Low permeability and metabolic instability are also common liabilities that hinder the therapeutic utility of this class of compounds [75]. To overcome such issues while simultaneously maintaining or enhancing bioactivity, medicinal chemists have modified quercetin and related natural products through the installation of additional functional groups, creating new synthetic flavonoids. In this section, we present some recent examples of the synthetic flavonoid approach that have translated to notable improvements in physiochemical and/or pharmacological advantages relative to the parent natural product. While most of the examples presented here did not arise specifically with wound healing in view, these instances nonetheless demonstrate general approaches to enhancing physiochemical and/or pharmacological properties that may be extended to a variety of therapeutic indications, including wound healing. 

**Figure 3:**
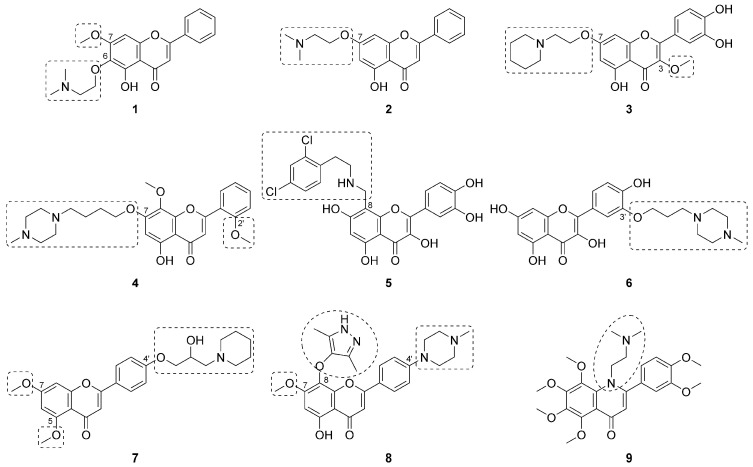
Synthetic flavonoids employing basic amine-containing groups as a means to improve physiochemical and/or pharmacological properties. Synthetic modifications to the parent natural product are highlighted with dashed-lines.

One of the more common strategies employed across synthetic flavonoid approaches representing a diverse array of indications and mechanisms of action involves the installation of flexible functional groups containing basic amines (Figure 3) [76,77,78,79,80,81,82,83,84]. Basic amines offer the opportunity to prepare salts, which can aid formulation and solubility in aqueous systems and create additional potential for favorable binding interactions with the pharmacological target(s) that may lead to increased potency. Depending on the chemical nature of the basic amine-containing group, the appendage may also increase lipophilicity relative to the parent compound, which can often improve the permeability of biological membranes.

Wu and coworkers synthesized negletein analog **1** (FZU-02,006), which contains a tertiary amine linked to the C^6^-oxygen via an ethylene tether [76]. It has been noted that negletein (7-*O*-methyl baicalein) has higher metabolic stability than baicalein [85]; thus, the methyl ether at C^7^ was maintained in **1**. The aqueous solubility of the HCl salt of **1** exceeded 1 mg/mL and was >25-fold more potent than baicalein and negletein in an assay measuring inhibition of proliferation of HL-60 leukemia cells. Compound **1** dose-dependently induced apoptosis in the same cell line, and mechanistic studies indicated inhibition of AKT-signaling as a potential mechanism of action for its anticancer activity [76]. Yang and coworkers prepared chrysin analog **2**, which possessed the same ethylene- linked tertiary amine as **1**, albeit at C^7^ rather than C^6^ [77]. The C^5^ hydroxyl and C^4^ ketone of **2** are proposed to function as a copper(II) chelator, which is thought to be key to its antioxidant and anti-Aβ_1-42_ aggregation activity. Further bolstering its potential application to Alzheimer’s disease (AD) therapy, compound **2** also functioned as an inhibitor of both acetylcholinesterase (AChE) and butyrylcholinesterase (BChE). While no physiochemical or pharmacokinetic characterization was reported for **2**, calculated properties were predictive of efficient blood–brain barrier (BBB) permeability [77]. 

In search of compounds with antioxidant and neuroprotective effects, Lee and coworkers synthesized **3**, a derivative of quercetin 3-*O*-methyl ether containing another ethylene-linked tertiary amine at C^7^ [78]. The HCl salt of 3 had more than 4000-fold higher aqueous solubility than quercetin 3-*O*-methyl ether while maintaining similar antioxidant activity in vitro. Moreover, in the middle cerebral artery occlusion (MCAO) rat model of cerebral ischemia, compound **3** was neuroprotective compared to vehicle as measured by significant reductions in corrected total infarct volume and percent edema [78]. Efforts to discover new compounds for the treatment of colon cancer by Sun and coworkers identified wogonin analog **4** (LZ-207). Compound **4** is another example with a tertiary-amine-containing group at C^7^; however, in this case an *N*-methylpiperazine was linked to the C^7^-oxygen via a saturated 4-carbon chain. A C^2′^ methyl ether was also present in **4**. The solubility of **4** was more than 125-fold higher than wogonin, and it inhibited viability and induced apoptosis in HCT116 colon cancer cells in vitro. Likewise, **4** dose-dependently suppressed nuclear translocation of the NF-κB transcription factor through interactions with upstream signaling pathways. Xenograft studies in nude mice bearing HCT116 tumors and treated with **4** demonstrated dose-dependent tumor growth inhibition [79]. Helgren and coworkers also used the A-ring for installation of an amine-containing group onto the quercetin scaffold; however, in this case the attachment was made via C^8^, and the analogs mainly consisted of secondary amines linked to aromatic rings via hydrocarbon chains [80]. These quercetin analogs were screened for activity against three drug-resistant malarial strains, and analog **5** was among the most active compounds, demonstrating 41- to 165-fold improved potency relative to quercetin. Despite the introduction of the highly lipophilic 2,4-dichlorophenethyl group, **5** exhibited nearly 4-fold improved aqueous solubility relative to quercetin, presumably due to the presence of the secondary amine [80].

Modification of the flavonoid scaffold as a strategy for improved physiochemical and/or pharmacological properties is not limited to the A-ring. For example, Mukherjee and coworkers prepared analogs of quercetin with polar functional groups on both the A- and B-rings and discovered B-ring-substituted analog **6**, which contains an *N*-methylpiperazine linked to the C^3′^-oxygen via a saturated 3-carbon chain [81]. These authors utilized a strategy focused on improving LogD_7_._4_, and **6** proved highly permeable in a Caco-2 bidirectional permeability assay with no evidence of efflux. Inhibition of cell proliferation in HCT-116 (human) and CT-26 (murine) colon cancer cells was greatly enhanced with compound **6** compared to quercetin at 96- and 88-fold, respectively. Mechanistic studies indicate that compound **6** induced oxidative stress in the HCT-116 cells and mediated apoptosis through the mitochondrial integrity pathway. Xenograft studies in BALB/c mice bearing CT-26 tumors demonstrated that compound **6** prolonged survival and dose-dependently reduced tumor volume [81]. Likewise, Chen and coworkers targeted C^4′^ of apigenin for installation of tertiary-amine-containing groups in their efforts to discover new therapeutics for chronic pancreatitis (CP) [82]. Analog **7** (HJC05100) contains a β-hydroxy amine group attached to the C^4′^-oxygen and methyl ethers at C^5^ and C^7^. The HCl salt of **7** demonstrated >38K-fold higher aqueous solubility than apigenin. Likewise, compound **7** enhanced potency in vitro relative to apigenin in a cell proliferation assay of pancreatic stellate cells and decreased fibrosis in an established mouse model of CP [82]. 

Wang and coworkers utilized a strategy focused on substitution of both the A- and B-rings of wogonin at C^8^ and C^4′^, respectively, in search of optimized cyclin-dependent kinase 9 (CDK9) inhibitors for the treatment of cancer [83]. Optimized analog **8** contains an *N*-methylpiperazine directly attached to C^4′^, an ether-linked 3,5-dimethyl-1*H*-pyrazol-4-yl group at C^8^, and a methyl ether at C^7^. Intrinsic aqueous solubility of **8** was more than double that of wogonin and more than 30-fold greater in a pH 4.5 buffered solution. Analog **8** demonstrated greater potency versus CDK9 than wogonin, with a superior selectivity profile versus CDK2. Likewise, **8** was a potent inhibitor of cell proliferation in a suite of 14 different tumor cell lines, and xenograft studies in nude mice bearing MV4-11 (leukemia) tumors demonstrated dose-dependent tumor growth inhibition following treatment with **8** without mortality or significant changes in body weight. Finally, use of the interior C-ring for installation of a tertiary-amine-containing group was also recently reported with nobiletin analog **9** [84]. Aqueous solubility of **9** was 280-fold higher than nobiletin in a pH 7.3 buffered solution and was effective at potentiating the effects of the anticancer agent paclitaxel in vitro, appearing to function as an inhibitor of the P-glycoprotein transporter and the PI3K/AKT pathway. Xenograft studies in nude mice bearing paclitaxel-resistant A549/T tumors demonstrated that **9** enhances the anticancer effects of paclitaxel, reducing tumor volumes without negatively affecting body weight [84].

Synthetic flavonoid strategies for improvement in pharmacological and/or physiochemical properties are not limited to the installation of basic amine-containing functional groups. The appendage of sugars to flavonoids through glycosylation has also been employed in multiple cases [86,87,88,89,90]. In fact, some flavonoids are found as *O*-glycosides in nature [75]; however, these *O*-glycosidic bonds are not always metabolically stable [88]. While solubility enhancement via installation of polar sugar groups is not surprising, such changes dramatically lower logP to levels considered unfavorable for passive permeation of lipophilic membranes such as the BBB. Still, transporters such as glucose transporter 1 (GLUT1) can sometimes bring such molecules into the brain via active transport [89].

**Figure 4:**
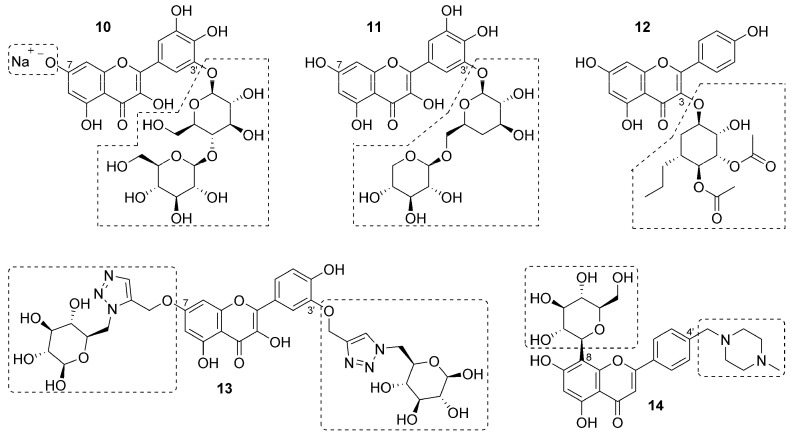
Synthetic flavonoids employing sugars as a means to improve physiochemical and/or pharmacological properties. Synthetic modifications to the parent natural product are highlighted with dashed-lines.

Zhu and coworkers prepared myricetin analog **10** via installation of a disaccharide at C^3′^ via a glycosidic bond and conversion to a sodium salt [86] (Figure 4). Aqueous solubility of **10** was excellent and more than 7000-fold higher than myricetin. Moreover, stability of **10** in rat plasma and rat liver microsomes was improved relative to myricetin as indicated by its longer half-life and reduced clearance in both systems. Evaluation of **10** in mice using a model of ulcerative colitis demonstrated its efficacy at reducing inflammation [86]. The same research group continued their optimization of disaccharide myricetin analogs in search of new hypoglycemic agents [87]. All disaccharide derivatives prepared and tested in this effort demonstrated α-glucosidase-inhibitory activity, which has relevance for the treatment of hypoglycemia, and **11** demonstrated 3-fold improved activity in vitro relative to myricetin.

Certain kaempferol derivatives glycosylated at C^3^ inhibit the p90 ribosomal s6 kinases RSK1/2, which has relevance to the treatment of certain types of cancers, including some triple-negative breast cancers. Li and coworkers have pursued carbasugar analogs of kaempferol in which the metabolically liability of the *O*-glycosidic bond has been eliminated by replacing the ring oxygen with a methylene unit as exemplified in **12** [88]. Carbasugar strategies have been utilized successfully in the design of marketed therapeutics, notably in the case of antiviral carbocyclic nucleoside drugs [89]. In addition to the methylene replacement of the ring oxygen, the carbasugar of **12** contains an *n*-propyl group as well as two acetate esters, modifications that raise lipophilicity relative to a traditional sugar. An asymmetric synthesis of the carbasugar moiety of **12** was developed beginning with D-(-)-quinic acid. Compound **12** demonstrated 6-fold enhanced potency versus RSK2 and in blocking proliferation of MCF-7 lung cancer cells in vitro compared to an *O*-glycoside lead. Likewise, **12** demonstrated improved bioavailability and efficacy in a mouse model of metastatic colonization relative to the same lead [88,90].

Antioxidant and neuroprotective effects of quercetin have relevance to the potential treatment of diseases characterized by neuronal injury and/or neurodegeneration. Given its poor aqueous solubility, low bioavailability, metabolic instability, and the fact that it does not readily cross the BBB [91,92,93], Wang and coworkers sought to improve such properties through glycosylation [94]. Desiring to avoid *O*-glycosides, the authors utilized click chemistry [95] to install glucose moieties at C^3′^ and C^7^ via triazole linkers to arrive at **13**. Analog **13** was effective at preventing hydrogen-peroxide-induced neurotoxicity in vitro and demonstrated enhanced neuroprotective effects in a rat model of cerebral ischemia (MCAO) compared to those observed with quercetin [93]. Recent studies have identified interactions between cellular prion protein (PrP^C^) and Aβ oligomers (Aβo) that contribute to the neurotoxicity observed in Alzheimer’s disease (AD). The flavonoid chrysin has been noted to disrupt Aβo-PrP^C^ and thus represents a potential starting point for the design of new AD therapeutics [96]. Matos and coworkers installed a *C*-glycoside at C^8^ and a flexible, basic amine-containing group at C^4′^ to arrive at **14**, a compound that exemplifies both strategies for synthetic flavonoids discussed herein. The solubility of **14** was enhanced compared to chrysin, and it dose-dependently disrupted interactions between Aβo-PrP^C^ in HEK cells. The C^8^ sugar was shown to be a key structure–activity relationship for this activity [96].

## 4. Future Outlooks

The identification of potential mechanisms of action and strategies to enhance bioavailability while retaining bioactivity through structural modification has substantially increased interest in the use of quercetin and related analogs for ocular wound healing. A substantial number of studies have demonstrated the therapeutic potential of quercetin and related analogs. With the vast majority of this research having been conducted in preclinical cell culture and animal models, questions regarding human efficacy and safety need to be addressed. While pharmacokinetic studies have been performed at the systemic level using dietary and dietary supplement forms of quercetin, there is a need to perform such studies on the enhanced and modified synthetic analogs. Improved bioavailability via encapsulation of quercetin and related flavonoids also remains an area for further research [97]. Determination of the optimal dosage depending on formulation and therapeutic application are still needed. Further, localized pharmacokinetic studies should be explored to better understand concentration and residence time at the site of action. Complementary pharmacodynamic studies could be used to further elucidate the mechanism of action and support targeted delivery through specific structural modifications. 

This may prove challenging. Quercetin was acknowledged by the Food and Drug Administration in 2010 as GRAS (Generally Recognized as Safe) for use as a dietary supplement, yet approval for any therapeutic purpose remains unseen. This demonstration will most likely require a concentrated effort in a specific application by a multidisciplinary team.

## 5. Conclusions

Quercetin and other flavonoids are bioactive compounds that have been found to exhibit therapeutic effects mediated via their antioxidant properties and activation of mitochondrial-associated factors, including AMPK and Sirtuin 1. As a phytoestrogen, quercetin is also known to bind cell-surface-bound and soluble forms of the estrogen receptors, thereby activating downstream signaling pathways, including estrogen response elements. Until recently, the limited solubility of quercetin and related flavonoids in aqueous solutions has blunted their therapeutic use in ocular applications. Strategies centered on the development of semisynthetic flavonoids substituted with groups designed to improve physiochemical and/or pharmacological properties have proven successful in a variety of preclinical settings and remain a growing area of research.

## Figures and Tables

**Table 1:** Selected preclinical evidence in animal models for the biological effects of quercetin in promoting tissue repair and anti-scarring properties in the anterior segment of the eye.

Biological Properties	Model System	Injury	Drug Treatment	Results	Ref.
Antifibrotic	C57BL/6J mice	Corneal epithelial and stromal debridement	Topical ocular application of 5 mM quercetin	Reduction in corneal opacity based on slit lamp	[36]
	New Zealand white rabbits	Corneal lamellar keratectomy	Topical ocular application of 5 mM quercetin	Reduction in corneal haze based on in vivo confocal microscopy	[36]
Antioxidative	Sprague- Dawley rats	STZ treatment to induce diabetes-associated complications	30–120 mg/kg quercetin administered i.g.	Reduced lens opacity based on slit lamp and decreased AGEs	[37]
	Sprague-Dawley rats	STZ treatment to induce hyperglycemia	90 mg/kg quercetin i.p.	Reduced lens opacity based on slit lamp and increased GSH production	[38]
Anti-inflammatory	NOD.B10.H2 mice	Injection of muscarinic receptor blocker followed by desiccation for 10 days to induce dry eye symptoms	Topical ocular application of 0.5% *w/v* quercetin	Increased tear production and reduced pro-inflammatory cytokine secretion	[39]
	WT C57BL/6 and T-cell-deficient C57BL/6 mice	Desiccation for 10 days and continuous injection of scopolamine hydrobromide to induce dry eye symptoms	Topical ocular application of 0.01% *w/v* quercetin	Improved ocular surface barrier function and decreased pro-inflammatory cytokine secretion	[40]

(Abbreviations: advanced glycation end products (AGEs); intragastric infusion (i.g.); intraperitoneal (i.p.); milligrams of quercetin per animal weight in kilograms (mg/kg); millimolar (mM); streptozotocin (STZ); weight to volume (*w*/*v*); wild-type (WT)).

## Data Availability

Not applicable.
